# Anxiety before extraction of impacted lower third molars

**DOI:** 10.4317/medoral.20105

**Published:** 2015-02-07

**Authors:** Beatriz Tarazona, Pablo Tarazona-Álvarez, David Peñarrocha-Oltra, Juan Rojo-Moreno, Maria A. Peñarrocha-Diago

**Affiliations:** 1 Associate Professor of Orthodontics. Department of Stomatology, Valencia University Medical and Dental School, Spain; 2 Master of Oral Surgery and Implantology. Department of Stomatology, Valencia University Medical and Dental School, Spain; 3 Full Professor of Psychiatry. Valencia University Medical and Dental School, Spain; 4 Full Professor of Oral Surgery, Department of Stomatology, Valencia University Medical and Dental School, Spain

## Abstract

Objetives: Assess levels of trait anxiety, state anxiety and dental anxiety before extraction of lower third molars and check the correlation and reliability of the scales used for the measurement of preoperative anxiety. Study Design: A prospective study of patients treated with extraction of a lower third molar between September 2010 to December 2010 was carried out. A total of 125 patients were included in the study. All of them were patients of the Oral Surgery and Implantology Department (Valencia University Medical and Dental School, Valencia, Spain). Before surgery, patients had to complete a preoperative protocol with 4 scales: the STAI-T (State-Trait Anxiety Inventory-Trait) for measuring trait anxiety, the STAI-S (State-Trait Anxiety Inventory-State) for measuring state anxiety, and DAS (Dental anxiety Scale of N. Corah) and APAIS (Amsterdam Preoperative anxiety and Information Scale) for measuring dental anxiety. Results: Patients undergoing extractions of an impacted lower third molar showed low levels of trait anxiety and moderate levels of state anxiety and dental anxiety. Higher levels of trait anxiety were obtained for older patients. Women had higher mean levels of dental anxiety and state anxiety that men with a statistically significant difference in STAI-S scales, DAS, and APAIS. Patients with higher trait anxiety and state anxiety showed higher levels of dental anxiety. A significant correlation (*p* ≤ 0.01) (*p* = 0.00) was found between the four scales used to measure anxiety. The scale showed higher correlation was STAI-S scale. The 4 scales showed high reliability (α of C.> 0.80). Conclusions: Patients with highest levels of trait anxiety and state anxiety, had more dental anxiety. The STAI-T, STAI-S, DAS and APAIS scales provided useful information about anxiety before the extraction of lower impacted third molars. The STAI-S is the scale with highest correlation and reliability.

** Key words:**Extraction of impacted lower third molars, preoperative anxiety, dental anxiety, trait anxiety, state anxiety.

## Introduction

Lower third molar surgery is related with dental anxiety (1). Before and during surgery, anxiety can affect patients physiologically and psychosomatically, increasing, for example, blood pressure affecting surgery and the incidence of complications. Therefore it is important to detect these patients before surgery (2). Trait anxiety is a permanent feature of the personality of each individual emotion and state anxiety refers to the emotional state of the human body when it is evaluated. Dental anxiety is a marked and persistent state of dental fear (3).

Several studies have revealed the relationship between trait anxiety, state anxiety and dental anxiety with surgery of impacted lower third molars. They found higher levels of anxiety in women (4-6). Lago-Méndez *et al*. (4) analyzed the relationship between trait anxiety, state anxiety and dental anxiety and he found trait anxiety was a predictor of dental anxiety, showing a statistically significant difference between men and women and trait anxiety. DAS (4,7,8) scale is the most used scale for measuring dental anxiety. However, few studies (5,6,9) have evaluated preoperative anxiety in dental patients by APAIS scale and its correlation with trait anxiety and state anxiety. All of them found a significant correlation between them as well as increased anxiety in women.

The aim of the study was to assess levels of trait anxiety, state anxiety and dental anxiety in patients who were undergoing lower third molars surgery and check the correlation and reliability of the scales used to measure the preoperative anxiety.

## Material and Methods

- Selection of the sample:

A prospective study was conducted from September 2010 to December 2010 in patients to undergo a single lower third molar surgery in the Oral Surgery and Implantology Department (Valencia University Medical and Dental School, Valencia, Spain).

145 patients were selected in the study. Only patients that properly completed each item of the 4 scales (STAI-T, STAI-S, DAS, APAIS) were included in the study. All patients were adults and signed an informed consent of the study. Patients who had taken any anxiolytic medication prior to the intervention were excluded previously. Of the 145 treated patients, 6 patients were excluded for lack of marked item , 4 for double marked item and 10 for had taken any anxiolytic medication prior to the intervention. Finally, 125 patients were included in the study.

Instruments for measuring anxiety :

The scales used for the measurement of anxiety were the STAI-Trait, for measuring trait anxiety and the STAI-State for measuring state anxiety as in others studies (10). The Dental Anxiety Scale of N. Corah (DAS) and Amsterdam Preoperative anxiety and Information Scale (APAIS) were used for measuring dental anxiety .

The STAI (10) consists of 40 questions, 20 questions for trait anxiety , STAI -T (State - Trait Anxiety Inventory- Trait ), and another 20 for state anxiety, STAI- S (State - Trait Anxiety Inventory -State). The scale consists of 4 possible answers with a score of 0 (none) to 3 (a lot) and the range of the values may be 0 to 60 points. To set the anxiety levels of the population were considered parameters of Kaakko *et al*. (11), who considered a non-anxious-patients patients until the first quartile and anxious-patients from second to fourth quartile.

The DAS (7,8) consists of 4 questions with 5 possible answers , in a ascending scale from 1 (relaxed) to 5 (almost physically ill), resulting in a range of values between 4 and 20. Patients were considered anxious forward from 12 points and patients as very anxious and with potentially dental phobic when the score is greater than 15 points.

The APAIS (9) consists of 6 items , 4 of them related to dental anxiety, (2 related to surgery and 2 related to anesthesia) and the other 2 items assess the need of information about the procedure and anesthesia. A scale of 1 (none) to 5 (a lot) was used and was completed in less than 2 minutes. The range of values obtained may vary from 6 to 30 points in the overall score, 4 to 20 in the dental anxiety and 2 to 10 in the need of information . Anxious patients are considered forward from 11 points.

- Data Collection 

Patients completed during the first visit the STAI-T (State-Trait Anxiety Inventory-Trait) (10) scale prior to receiving any information about treatment, to measure trait anxiety treatment.

After filling the STAI-T scale, all patients were explained the anesthetic and surgical procedure, possible complications, and they were collected a informed consent and the acceptance of participation in the study. General patient data (age and sex) were recorded.

On the day of surgery in the waiting room, they completed the STAI-S questionnaire (State-Trait Anxiety Inventory-State) (11) for measuring state anxiety, and scale DAS (Dental Anxiety Scale) (7,8) and APAIS (Amsterdam Preoperative anxiety Scale and Information) (10) for measuring dental anxiety.

- Statistical analysis.

All data were collected in a protocol for each patient. They were stored in a data base and then processed statistically. Analyses were performed using SPSS 15.0 (SPSS Inc., Chicago, IL) for Windows. To know the relationship between different variables and anxiety Student t test was used and the Mann Whitney U-Test, the choice of test statistic appears in the description of the results. Finally, a Cronbach’s alpha test was performed to establish the homogeneity of the scales and a Pearson correlation to establish correlations between the scales.

## Results

The sample consisted of 125 patients, 53 men and 72 women. The mean age of patients was 24.9 years (range 18 to 52 years); 24.5 years for men and 25.2 years for women.

For trait anxiety (STAI-T scale), the number of non-anxious patients was 80 and 45 anxious patients. With he STAI-S (state anxiety) scale, there were found 58 patients non-anxious patients and 67 anxious. For dental anxiety scale (DAS), the number of anxious patients was 48 patients and 77 non-anxious patients. Finally, in the APAIS scale, the number of anxious patients was 55 and the number of non-anxious patients was 70. In  Table 1 and  Table 2, we can see the distribution and relationship of positive and negative trait and state anxiety, and dental anxiety.

**Table 1:**
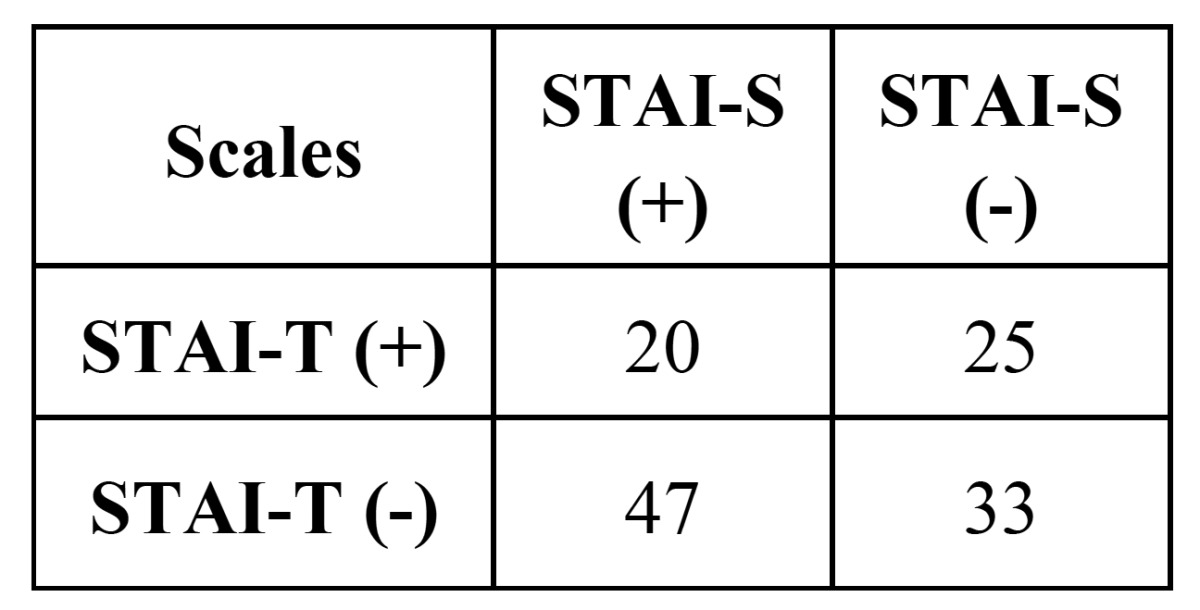
Relationship of the presentation of anxiety as state and trait anxiety in the sample (χ2, *p*=0,002).

**Table 2:**
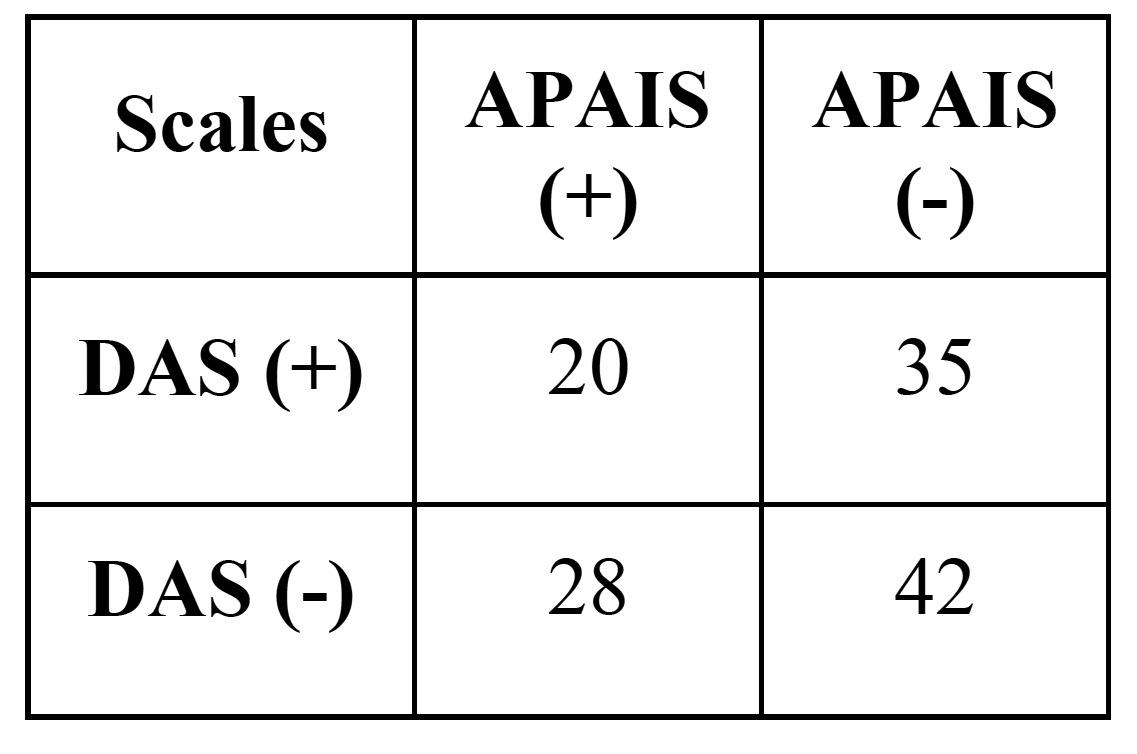
Relationship of the presentation of dental anxiety in the sample (χ2, *p*=0,002).

A statistically significant relationship (*p* = 0.034) between age and trait anxiety in the STAI-T scale was observed , showing higher trait anxiety with increasing age but in the others scales there was no statistically significant relationship (*p*>0.05). Regarding gender, higher values are always observed for women in the 4 scales used ( Table 3), finding in the STAI- S, DAS and APAIS scales a statistically significant difference (*p*≤0.05) between gender, but not in the STAI-T scale for measuring trait anxiety .

**Table 3:**
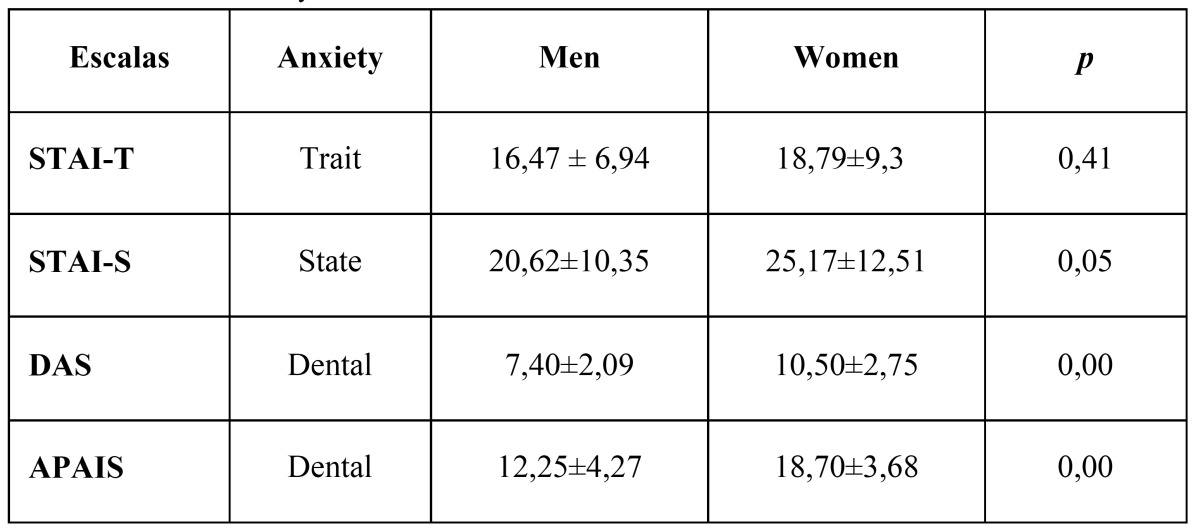
Levels of anxiety.

**Figure 1:**
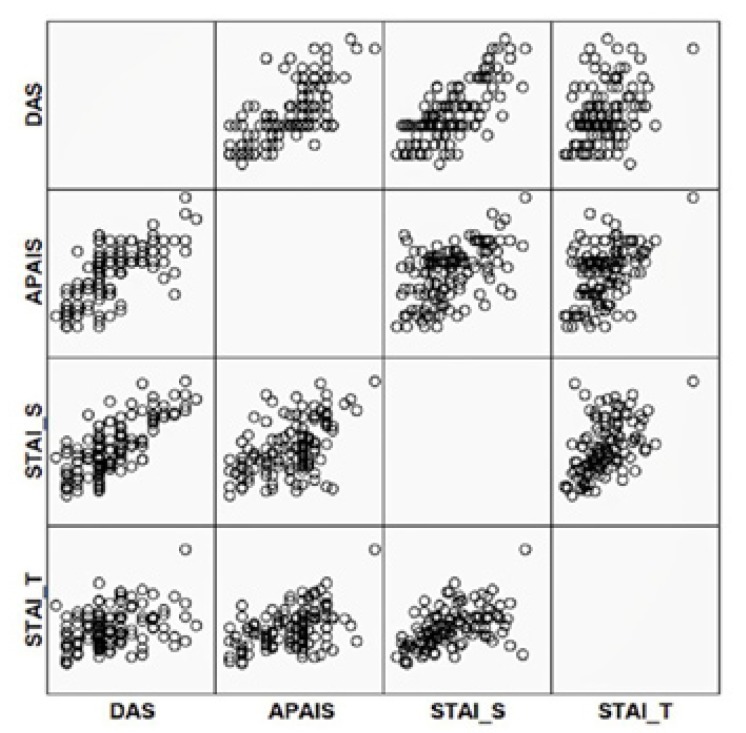
Correlation between the 4 scales.

In figure 1, we could observe a statistically significant correlation (*p* ≤ 0.01) with a very similar response between 4 scales in the scatter plot. We could evidence the same degree of compatibility between the scales used ( Table 4), obtaining the highest values between STAI-S scale and DAS, and between APAIS scale and DAS.

**Table 4:**
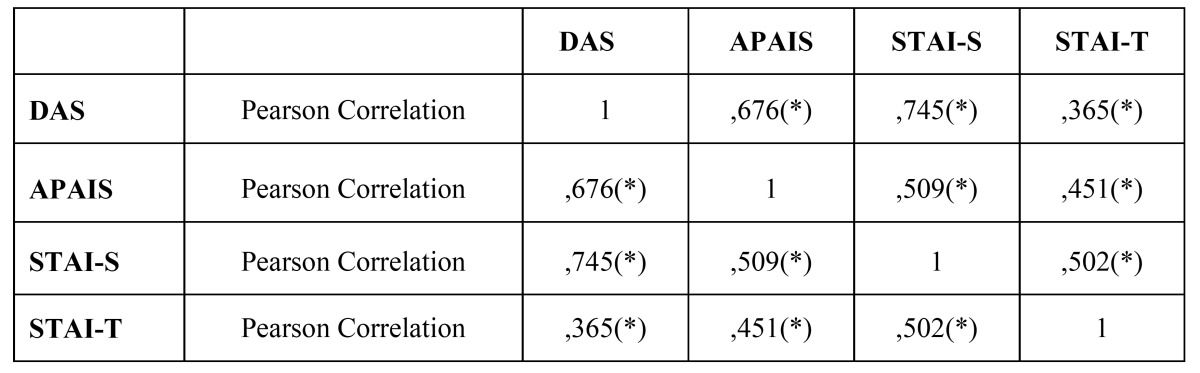
Correlation between scales.

Also high reliability of the 4 scales (α of C.> 0.80) was obtained, with a Cronbach Alpha value of 0.85 in the STAI-T scale, 0.93 for the STAI-S, 0.87 for the DAS and 0.84 for APAIS. The scale of state anxiety (STAI-S) was the most reliable.

## Discussion

There are different scales for measuring trait anxiety, state anxiety and dental anxiety (6,7,9). The most important scale in Psychology for measuring the state anxiety and trait anxiety is STAI (10) scale. Nevertheless, DAS (4,7,8) scale is the most used scale for measuring dental anxiety. There are few studies that use the APAIS (5,9) scale for the measurement of preoperative anxiety in surgery of impacted lower third molars , since it was created in 1996.

A statistically significant relationship between age and trait anxiety was found using the STAI-T (*p* = 0.034) scale. The levels of trait anxiety were increased with age, but there was no relationship to dental anxiety and state anxiety. These results have been observed by other authors (12,13). In contrast, Hagglin *et al*. (14) found a decrease of dental anxiety with increasing age, observed in a longitudinal study of 28 years with 1622 women in a Swedish population of Göteborg. Participants were examined at 6, 12, 24 and 28 years, with higher levels in the first visit. For authors, dental anxiety produced by the dentist, as well as other specific phobias, decreased with age.

In most studies (3,4,5,14) including this study, it has been observed higher levels of dental anxiety in women compared to men, there are a significant discrepancy between gender. In a study (15) conducted on adolescents between 12 and 18 years old, the results were the same, they found higher levels of anxiety in girls. Regarding general anxiety, Hakeberg *et al*. (16) found higher levels of general anxiety in women than in men, but other studies have found no differences between gender (17,18). Some studies (19) argue that these differences in anxiety levels between gender may be attributable to the different thresholds for pain between men and women. For other authors (20,21), the differences are due to the fact that women express more freely their fears against men.

If we focus on the different scales for measuring anxiety, Lago-Méndez *et al*. (4), Kvale *et al*. (22) and Hakeberg *et al*. (16) obtained, as in this study, a significant correlation between dental anxiety and state anxiety (between STAI-S and DAS) scales. Kvale *et al*. (22) concluded that the DAS scale for measuring dental anxiety is as reliable as a STAI-S scale for measuring anxiety state. However, other authors (18,23) found no significant relationship between the two scales. We also found as several studies (23,24) a statistically significant correlation (*p* <0.05) between the scale for measuring dental anxiety (APAIS). Therefore, 4 questions related to the measurement of anxiety are comparable to the 30 item of STAI-S scale.

Our results indicate that we can find greater anxiety for possible complications in the case of older people, within the range 18-52 years old, and especially if they are female. However, the determination of whether this particular vulnerability is defined in operative or postoperative complications are not the objectives of this study.

Regarding reliability, Kvale *et al*. (22) obtained a high reliability of α Cronbach’s greater than 0.95 for both the STAI-S scale and the DAS scale, the same as our study a 0.93 obtained for S STAI scale. Moreover Moerman *et al*. (9) observed a α Cronbach’s of 0.86 in the APAIS scale whereas the present study obtained a value of 0.84. These value may have been due to the homogeneous sample.

## Conclusions

Patients with highest levels of trait anxiety and state anxiety, had more dental anxiety. The STAI-T, STAI-S, DAS and APAIS scales provided useful information about anxiety before the extraction of lower impacted third molars. The STAI-S is the scale with highest correlation and reliability.
